# Probing Difference in Binding Modes of Inhibitors to MDMX by Molecular Dynamics Simulations and Different Free Energy Methods

**DOI:** 10.1371/journal.pone.0141409

**Published:** 2015-10-29

**Authors:** Shuhua Shi, Shaolong Zhang, Qinggang Zhang

**Affiliations:** 1 School of Science, Shandong Jianzhu University, Jinan, China; 2 College of Physics and Electronics, Shandong Normal University, Jinan, China; University of Calgary, CANADA

## Abstract

The p53-MDMX interaction has attracted extensive attention of anti-cancer drug development in recent years. This current work adopted molecular dynamics (MD) simulations and cross-correlation analysis to investigate conformation changes of MDMX caused by inhibitor bindings. The obtained information indicates that the binding cleft of MDMX undergoes a large conformational change and the dynamic behavior of residues obviously change by the presence of different structural inhibitors. Two different methods of binding free energy predictions were employed to carry out a comparable insight into binding mechanisms of four inhibitors PMI, pDI, WK23 and WW8 to MDMX. The data show that the main factor controlling the inhibitor bindings to MDMX arises from van der Waals interactions. The binding free energies were further divided into contribution of each residue and the derived information gives a conclusion that the hydrophobic interactions, such as CH-CH, CH-π and π-π interactions, are responsible for the inhibitor associations with MDMX.

## Introduction

Recently, due to key roles in maintaining the integrity of the genome, the tumor suppressor protein p53 has been paid an extensive attention[1]. P53 protein can coordinate the cellular response to DNA damage by inducing cell cycle arrest or apoptosis[2]. Active p53 can efficiently hold back tumor’s growth and protect human health[3]. The previous studies suggested that the inactivation of p53 is tightly related with human cancer, which mainly arises from either point mutations in TP53 gene or functional inhibition by negative regulators MDM2 and MDMX[4–8]. The data from clinical treatments proved that over-expressions of two proteins MDM2 and MDMX were found in ~50% of all cancer patients around the globe, which significantly influences the wild-type function of p53[9–11].

MDMX is also named as MDM4 and highly homologous to MDM2. It is another significant negative regulator of p53[4, 12]. Three key residues (Phe19´, Trp23´ and Leu26´) can form direct interactions with MDM2/MDMX[13–15]. Although MDMX has overall similar structure to MDM2, it does not possess the capability of ubiquitin ligase and its expression level is not related with p53[16–19]. Several previous experimental studies also demonstrated that inhibition effect of current inhibitors on MDMX is weaker than that on MDM2[7, 20–23]. Thus, it is essential to further reveal the interaction mechanism of inhibitors with MDMX for development of potent inhibitors rescuing the wild-type function of p53.

Up to now, many researches have focused on development of inhibitors restoring the p53 function[24–29]. Lots of existing inhibitors displayed strong inhibition ability on the p53-MDM2 interaction, but they can not efficiently control the association with MDMX. Detailed clarification of the binding modes of peptide and non-peptide inhibitors to MDMX can contribute valuable information on the structure-affinity relationship of the MDMX binding compound, which is helpful for designs of efficient inhibitors. Joseph et al. applied MD simulations and binding free energy calculations to reveal the cause of the binding difference of p53 and nutlin to MDM2 and MDMX[30]. Li et al. explored the inhibition mechanism of inhibitors on the p53-MDM2/MDMX interaction by performing systematic mutational analysis on p53 and peptide inhibitor PMI[31]. Chen et al. combined MD simulations and computational alanine scanning method to successfully investigate the difference in binding modes of inhibitors to MDM2 and MDMX[32]. Due to high flexibility of hydrophobic cleft of MDMX, further insights into the binding mode and conformational changes of MDMX induced by inhibitor bindings is of importance for the designs of potent inhibitors interrupting the p53-MDMX interaction.

**Fig 1:**
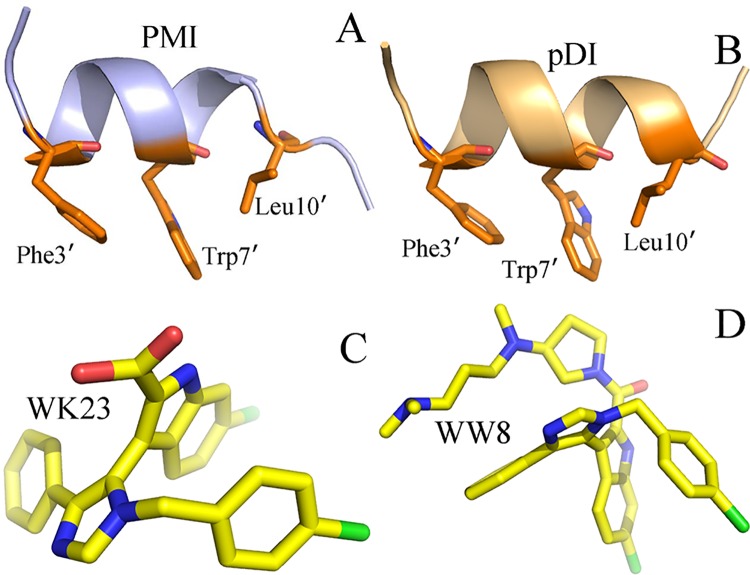
Molecular structures of the inhibitors: (A) PMI, (B) pDI, (C) WK23 and (D) WW8.

Two peptide inhibitors PMI and pDI, together with two non-peptide inhibitors WW8 and WK23, were picked out to study their binding modes to MDMX. PMI is a peptide inhibitor (TSFAEYWNLLSP) designed by Pazgier et al. This inhibitor not only structurally shares three common residues (Phe3´, Trp7´ and Leu10´) with p53, but also has a strong inhibition ability scaled by K_d_ value of 4.15 nM[33]. pDI is another peptide inhibitor (LTFEHYWAQLTS) contributed by Phan et al., which shows a weak inhibition potency on MDMX with IC50 value of 550 uM and structurally also shares three key residues with p53[34]. WW8 and WK23 are two non-peptide inhibitors studied by Popowicz et al. and their Ki values are 11 and 36 uM[14], respectively. The structures of these inhibitors were displayed in Fig 1. Although pDI and PMI share three same key residues with p53 and the structures of WW8 and WK23 are also highly similar, their binding affinities to MDMX are greatly different. Thus it is of significance to understand the cause leading to these difference of binding abilities for designs of small molecule inhibitors blocking the p53-MDMX interaction.

MD simulations and principal component (PC) analyses have showed huge potentiality in probing the interaction mechanisms of inhibitors with proteins and conformational changes of proteins[35–50]. MM-GBSA method has been successfully used to study inhibitor-protein binding modes in many macromolecules[51–56]. Thus, in this study, MD simulations, PC analyses, MM-GBSA and solvated interaction energy (SIE) methods were integrated to investigate the difference in binding modes of inhibitors to MDMX and reveal the conformational changes of MDMX produced by inhibitor bindings.

## Methods

### Starting structures

The crystal structures of three compounds with pDI, PMI and WW8 taken from the protein data bank were used as the starting model of MD simulations and their corresponding PDB entries are 3EQY, 3JZO and 3LBJ, respectively[14, 33, 34]. Because the crystal structure of the WK23-MDMX compound is not available, it can be got by modifying the WW8-MDMX structure. The Leap program included in Amber was employed to connect all missing hydrogen atoms with the heavy atoms of MDMX[57]. During the initialization of the simulated structures, all water molecules originating from the crystal structures were retained. The force field parameters of proteins and crystal water molecules were gained by using the FF03 force field[58]. The structures of WK23 and WW8 inhibitors were minimized at semi-empirical AM1 level and BCC charges were assigned to them by using the Antechamber program in Amber package. The force field parameters of WK23 and WW8 were generated by using the general Amber force field (GAFF)[59]. To neutralize the charge of each system, an appropriate number of chloride counterions were added to four inhibitor-MDMX compounds. To reflect the solvent environment of proteins, an octahedral periodic box consisting of TIP3P water molecules with a buffer of 12.0 Å was adopted to solvate the initialized system.

### MD simulations

To relieve bad conformation between atoms, energy minimization and MD simulations were performed on all initialized systems by using the Amber dynamics program. Firstly, the starting models were minimized in 3000 steps with constraint on the compound using a harmonic restriction of a strength of 100 kcal·mol^-1^·Å^-2^. Next, full minimization of another 3000 ps without any restraints were run[32, 60]. Then, the temperature of system was slowly heated from 0 to 300 K in 500 ps. To achieve a reliable stability, the system endured an equilibration of another 500 ps at 300K. Finally, 60-ns MD simulations without restriction were conducted on each system at 1 atm and 300 K to obtain a stable trajectory for the following post-processing analysis. The covalent bonds connecting with hydrogen atoms were restrained by using the SHAKE method so that the time step of 2 fs was used during MD simulation[61]. The Particle Mesh Ewald (PME) method was adopted to compute the long-range electrostatic interaction[62, 63]. The cutoff distances for the non-bonded interactions were set to 10 Å.

### Solvated interaction energy method

The SIE method developed by Naim et al. was usually applied to predict the binding free energies of inhibitors to proteins in many life systems by using the following equation[64]:

$$\Delta G_{bind}(\rho ,D_{in},\alpha ,\gamma ,C)=\alpha \times [E_{c}(D_{in})+\Delta G^{R}+E_{vdW}+\gamma \cdot \Delta MSA(\rho )]+C$$ (1)

where *E*_*c*_ is the intermolecular Coulomb interactions in the bound state and the parameter *D*_*in*_ is the solute interior dielectric constant. The term *E*_*vdW*_ represents the van der Waals interaction energies of inhibitors with proteins. These two terms can be computed by using the Amber molecular force field FF03[58]. The term Δ*G* ^*R*^ reflects the reaction field energy difference generated by inhibitor associations, and was obtained by solving the Poisson equation using the boundary element method BRI BEM[65, 66] and a variable-radius solvent probe[67]. The fourth term *γ* • Δ*MSA*(*ρ*) is related with the change of the molecular surface area induced by inhibitor associations and the parameter *γ* is the molecular surface area coefficient. The parameters *α* and *C* are the global proportionality coefficient related to the loss of conformational entropy upon binding and the constant, respectively. All parameters involved in the current calculations are set to α = 0.1048, *D*_*in*_ = 2.25, *D*_*in*_ = 2.25, *ρ* = 1.1, γ = 0.0129 kcal/(mol·Å2) and *C* = −2.89 kcal·mol^−1^, respectively[64, 68, 69]. Although the coefficients for SIE were parameterized using the AMBER ff99SB force field[70], our previous studies on the interaction mechanism of inhibitors with MDM2 based on FF03 force field proved that these coefficients of the SIE method can also produce rational results in binding free energy predictions[71]. The SIE method has obtained great success in insight into the structure-affinity relationship of several inhibitor-protein binding systems[69, 72]. Thus, the SIE method was adopted to evaluate the binding abilities of inhibitors to MDMX by using the program Sietraj and 200 snapshots taken from the last 20 ns of MD trajectory at an interval of 100 ps[68].

### MM-GBSA calculations

MM-GBSA method[73] can be also used to predict the binding free energies of inhibitors to proteins. The previous studies suggested that this method has obtained success in studying the inhibition mechanisms of the p53-MDM2/MDMX association[32, 47, 53]. Thus, MM-GBSA method were implemented to compute the binding free energies of the current four inhibitors to MDMX based on the following empirical equation.

$$\Delta G=\Delta E_{ele}+\Delta E_{vdw}+\Delta G_{pol}+\Delta G_{nonpol}-T\Delta S$$ (2)

in this equation, the first two terms Δ*E*_*ele*_ and Δ*E*_*vdw*_ represent the electrostatic and van der Waals interactions, respectively and were calculated by using the Amber molecular mechanics force field FF03. The term Δ*G*_*pol*_ is the polar contribution to solvation free energy, which was computed by using the modified GB model developed by Onufriev et al.[74]. The fourth term (ΔG_nonpol_) in Eq 2 is the non-polar solvation energy and was computed by using the empirical Eq 3

$$G_{nonpol}=\gamma \times SASA+\beta$$ (3)

in the current calculation, the parameters *γ* and *β* were assigned as 0.0072 kcal·mol^−1^·Å^−2^ and 0.0 kcal·mol^−1^, respectively[75]. The last term (*T*Δ*S*) is the entropy contribution and comes from the changes in the translational, rotational and vibrational degrees of freedom induced by inhibitor bindings, which is calculated by using the normal-mode analysis[76]. In this work, 200 snapshots taken from the last 20 ns of MD trajectory at an interval of 100 ps were used for MM-GBSA calculation and 50 snapshots from the last 20 ns of MD simulations at an interval of 400 ps for the normal-mode analysis.

### Internal dynamics

The cross-correlation analysis is a significant tools to study the conformation change and internal dynamics of proteins. To probe the changes of internal dynamics of MDMX induced by inhibitor associations, the cross-correlation matrix *C*_*ij*_ for *C*_*α*_ atoms *i* and *j* can be constructed by using the following equation[77].

$$c_{ij}=\frac{<\Delta r_{i}\cdot \Delta r_{j}>}{{\left(\Delta r_{i}^{2}\cdot \Delta r_{j}^{2}\right)}^{1/2}}$$ (4)

in which Δ**r**_*i*_ describes the displacement of the *i*th atom relative to its average position. The angle bracket indicates an ensemble average over the frames extracted from the equilibrated MD trajectory. The values of *C*_*ij*_ fluctuate in the range from -1 to 1. The positive value of *C*_*ij*_ indicates a correlated motion between C_α_ atoms, while the negative one implies an anti-correlated motion.

## Results and Discussions

### Equilibrium of dynamics simulations

**Fig 2:**
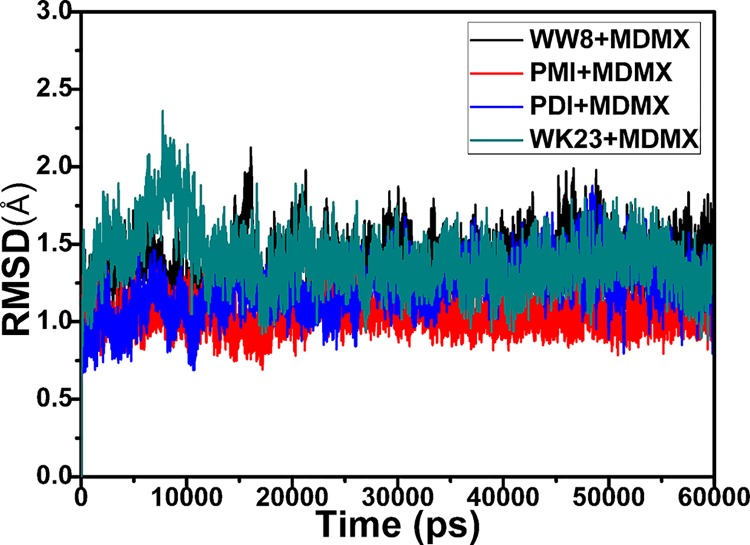
RMSD values of the backbone atoms relative to the initial minimized structures as functions of simulation time.

The root-mean-square deviations (RMSD) of backbone atoms in MDMX relative to the initial minimized structures through the phase of MD simulations were calculated and depicted in Fig 2. These RMSD values can be used to evaluate the reliability of MD simulation equilibrium. Fig 2 shows that four compound systems with inhibitors have achieved the equilibrium in ~15 ns of MD simulations. The calculated RMSD values of four compounds with PMI, pDI, WK23 and WW8 are 1.12, 1.30, 1.33 and 1.45 Å, respectively. The fluctuating range of these RMSDs is smaller than 0.55Å. Therefore, a conclusion is drawn from this result that the trajectories of MD simulations of four systems are reliable. Fig 2. indicates that the RMSD values of the compounds with pDI, WK23 and WW8 are higher than that of the PMI-MDMX compound, which implies that the restriction of these three inhibitor bindings on MDMX may be weaker than that of PMI.

### Dynamics of MDMX

**Fig 3:**
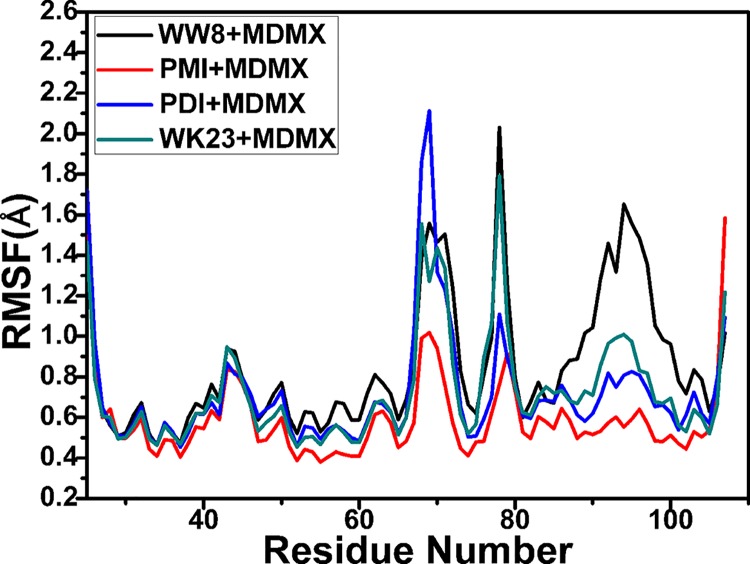
RMSF of C_α_ atoms in MDMX through the equilibrium phase of MD simulations.

Root mean square fluctuation (RMSF) is generally applied to evaluate the fluctuation of a specific residues relative to a reference structure. To reveal the flexibility of MDMX, the averaged RMSF values of *C*_*α*_ atoms were computed by using the trajectories after the MD equilibrium (Fig 3). Fig 3 demonstrates that MDMX restricted by four inhibitors result in similar fluctuation tendency, but obvious difference of RMSF values are also identified near the residues 70, 78 and 86–105, which suggests that the interactions of the inhibitors with MDMX produce different restrictions on the motions near these residues. The above difference of RMSF values may imply the changes of internal dynamics and interaction intensities.

**Fig 4:**
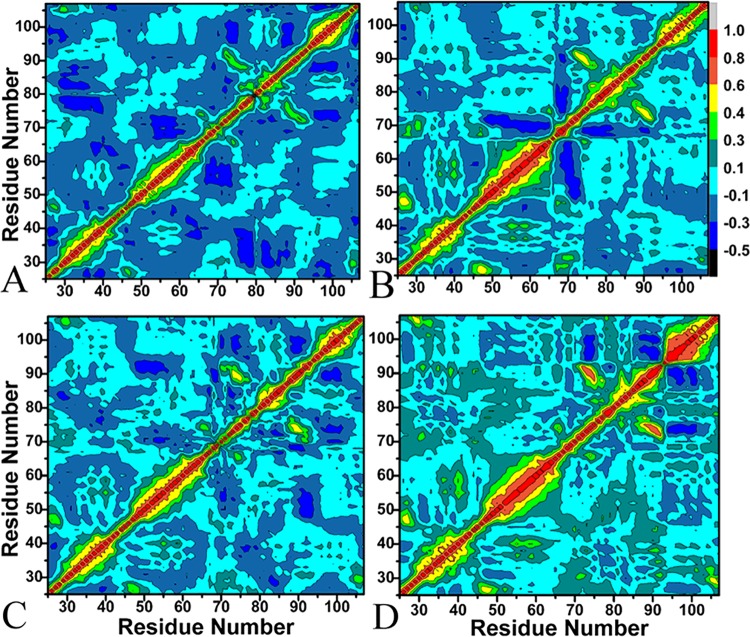
Cross-correlation matrices of the fluctuations of the coordinates for C_α_ atoms of MDMX around their mean positions during the equilibrium phase of simulations. The extents of correlated motions and anticorrelated motions are color-coded for four binding complexes: A for PMI-MDMX, B for pDI-MDMX, C for WK23-MDMX and D for WW8-MDMX.

To further discover the impact of inhibitor associations on internal dynamics of MDMX, the cross-correlation matrices describing the fluctuations of *C*_*α*_ atoms were computed (Fig 4). Fig 4 reveals that there are few highly correlated motions except for the diagonal regions (red and yellow). However, the associations of these four inhibitors with MDMX generate obvious effect on the motion modes of several regions in MDMX.

Fig 4A shows that the presence of PMI induces obvious anti-correlated motions in MDMX, which corresponds to the domain labeled by the dark blue color. The PMI association leads to strong anti-correlated motions of the domain consisting of the residues 65–72 relative to the domain formed by the residues 51–58. The PMI association also produces obvious anti-correlated motions of the domain (the residues 75–88) relative to the domain (the residues 27–40). Additional anti-correlated motions also appear in the diagonal region consisting of the residues 90–107. Except for the above anti-correlated motions, slight correlated motions can be found in the diagonal region indicated by yellow.

The comparison with the PMI-MDMX compound shows that the associations of pDI, WK23 and WW8 with MDMX lead to obvious changes of internal motions (Fig 4B–4D). The presence of these three inhibitors strengthen not only the correlated motions in the domain (the residues 49–65), but also the correlated motions of the inter-domain between the residues 88–93 and 71–76. For the WW8-MDMX compound, the association of WW8 induces the increase of correlated motions in the diagonal region consisting of the residues 94–107 (red). The bindings of inhibitors WK23 and WW8 to MDMX result in the disappearance of anti-correlated motions of the inter-domain between the residues 65–72 and 51–58, while the pDI binding increases this anti-correlated motions. Finally, the anti-correlated motions between the residues 75–88 and 27–40 disappear due to the restriction of these three inhibitors. The above results prove that different inhibitor bindings induce the changes of internal dynamics in MDMX during MD simulations, which reflects that the flexibility of MDMX is large and inhibitor bindings produce the different dynamic behavior in MDMX. The analysis reported here basically agrees with the previous RMSF analysis.

**Fig 5:**
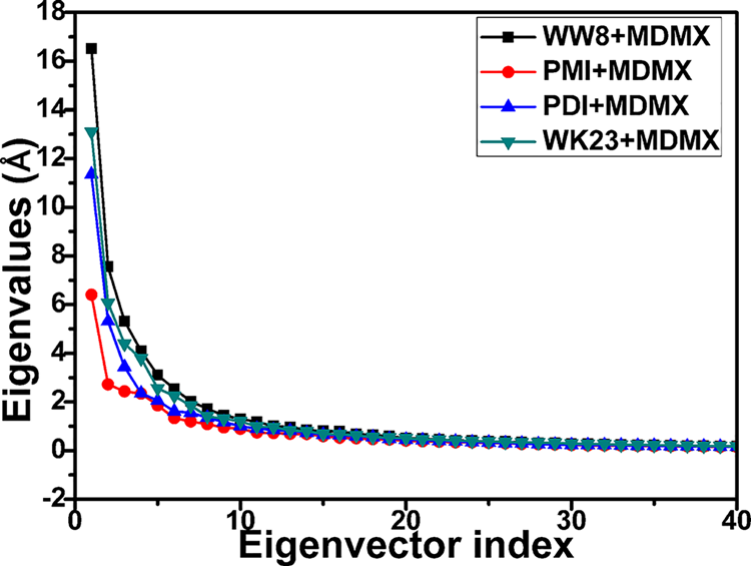
Comparison of the eigenvalues plotted against the corresponding eigenvector index obtained from the covariance matrix of C_α_ atoms constructed from the equilibrium of MD simulations.

To evaluate the overall effect of inhibitor associations on the flexibility of MDMX, the eigenvalues were obtained by diagonalizing the covariance matrix constructed by the atomic coordinate[78, 79] and the information was displayed in Fig 5. The first several eigenvalues indicate the concerted motions of residues. It is observed that these eigenvalues quickly reduce in amplitude to reach a number of constrained and more localized movement. The first six principal components contribute up to 58.2%, 59.8%, 61.4% and 69.4% in the total motions for the PMI-MDMX, pDI-MDMX, WK23-MDMX and WW8-MDMX compounds, respectively. This result suggests that the bindings of different structural inhibitors produce different effects on the concerted motions of MDMX. The above analyses basically agree with the previous studies[29, 30, 60].

### Binding free energy calculations

To clarify the binding mechanism of inhibitor bindings to MDMX, binding free energies were computed by using MM-GBSA method based on MD trajectories. The computed results were given in Table 1. One can see from Table 1 that the binding free energies of PMI, pDI, WK23 and WW8 to MDMX are -13.3, -10.7, -8.4 and -8.9 kcal·mol^−1^, respectively. It is encouraging that the rank of our predicted binding free energies agrees well with the experimentally determined one. As shown in Table 1, although the predicted binding free energies are about -2.2 kcal·mol^−1^ higher than the corresponding experimental values, a good correlation was observed between our predicted values and the experimental ones. Although Cheng et al also computed the binding free energies of PMI and WW8 to MDMX, their predicted results is much higher than the experimental values. Chen et al. also evaluated the association ability of WK23 with MDMX, but their predicted value is 8.81 kcal·mol^−1^ stronger than the experimental value.

**Table 1:** Binding free energies calculated by MM-GBSA method (kcal·mol^−1^).

Energy^a^	PMI+MDMX	pDI+MDMX	WK23+MDMX	WW9+MDMX
ΔE_ele_	-136.2±7.6^b^	-141.9±8.4	-32.9±6.4	-53.1±6.8
ΔE_vdw_	-64.8±2.1	-55.1±2.1	-36.0±1.9	-37.1±1.4
ΔG_nonpol_	-9.5±0.1	-8.5±0.3	-5.1±0.1	-5.5±0.2
ΔG_pol_	158.3±8.7	161.9±10.2	46.7±6.8	67.1±7.2
-TΔS	38.9±1.2	32.9±1.6	18.9±1.1	19.7±1.5
ΔG_bind_	-13.3	-10.7	-8.4	-8.9±
ΔG^exp^	-10.1	-8.5	-6.1	-6.8±

^a ^Component: ΔE_ele_: electrostatic energy in the gas phase; ΔE_vdw_: van der Waals energy; ΔG_nopol_: non-polar solvation energy; ΔG_pol_: polar solvation energy; -TΔS: total entropy contribution; ΔG_bind_ = ΔG_gas+sol_−TΔS.

^b^ The signs “±” represent standard errors determined by using $std=\sqrt{\frac{\sum {\left(x_{i}-\bar{x}\right)}^{2}}{N-1}}$.

Table 1 shows that the van der Waals interaction (Δ*E*_*vdw*_) and non-polar solvation energy (Δ*G*_*nonpol*_) favor the association of inhibitors with MDMX. Although the electrostatic interactions (Δ*E*_*ele*_) provides a favorable force to the inhibitor bindings, it completely screened by the polar solvation energy to lose its advantage. Furthermore, it is noted that van der Waals interaction is much stronger than non-polar solvation energy. Thus, a conclusion was obtained that the van der Waals interactions mainly control the bindings of these inhibitors to MDMX.

To complement our results predicted by MM-GBSA method, the SIE method was also employed to compute the association affinity of these inhibitors to MDMX (Table 2). Table 2 suggests that the binding free energies of PMI, pDI, WK23 and WW8 to MDMX are -10.71, -9.92, -6.89 and -7.09 kcal·mol^−1^, respectively. Moreover, the rank of the predicted binding free energies is also in good accordance with the experimental determined one. The above results from two methods show that the current free energy analysis is reliable.

**Table 2:** Binding free energies calculated by SIE method (kcal·mol^−1^).

^a^Energy	PMI+MDMX	pDI+MDMX	WK23+MDMX	WW8+MDMX
ΔE_vdW_	-61.11±2.08^b^	-58.54±2.41	-36.57±1.11	-37.82±1.44
ΔE_c_	-64.99±7.48	-66.67±9.82	-15.84±2.87	-21.58±3.68
γ·ΔMSA	-11.67±0.58	-10.84±0.61	-6.29±0.12	-6.91±0.42
ΔG^R^	63.13±6.73	68.98±7.53	20.52±3.05	26.23±3.45
C	-2.89±0.0	-2.89±0.00	-2.89±0.0	-2.89±0.00
ΔG_bind_	-10.71±0.47	-9.92±1.97	-6.89±0.30	-7.09±3.54

^a^ΔE_vdW_ represent the van der Waals interaction energy between inhibitors and MDMX

ΔE_c_ is the intermolecular Coulomb interaction energy; γ·ΔMSA corresponds to the hydrophobic interaction related with the molecular surface area upon binding; ΔG^R^ is the reaction field energy change between the bound and free states

^b^The signs “±” represent standard errors.

By comparing the results from two methods, it is seen that the ranks of binding free energies predicted by two methods agree well with the experimental one, but the binding free energies predicted by the SIE method are quantificationally closer to experimental values than those by MM-GBSA method. Based on these two calculations, a common conclusion was derived that the van der Waals interactions are responsible for the bindings of inhibitors to MDMX. By comparison of free energy components, it is found that two peptide inhibitors PMI and pDI can form more interaction contacts than two non-peptide inhibitors. The above analyses basically agree with the previous studies[30, 32, 60].

### The structure-affinity relationship

**Fig 6:**
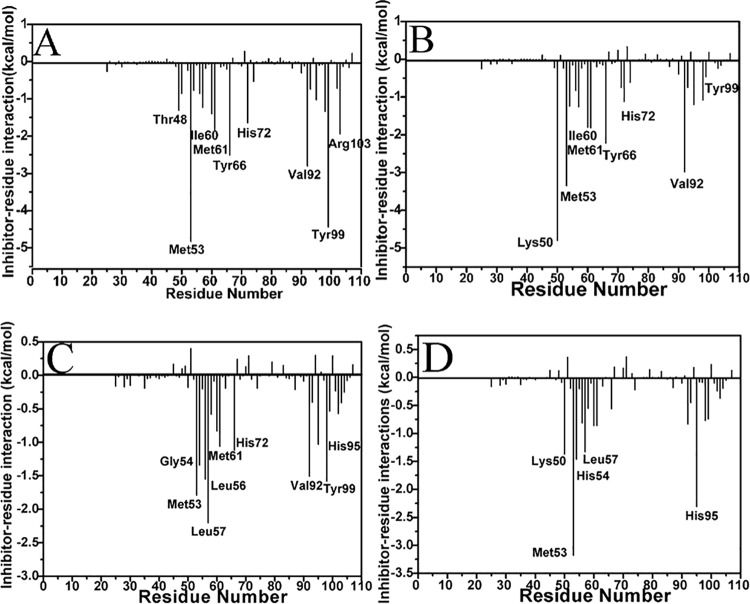
The interaction spectra of the separate residues of MDMX with the inhibitors: (A) PMI, (B) pDI, (C) WK23 and (D) WW8.

**Fig 7:**
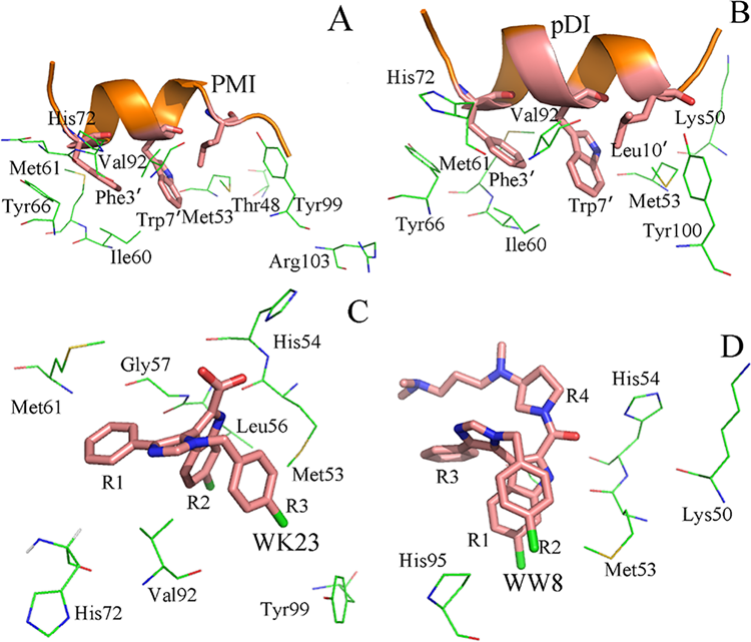
Geometries of key residues, which produce favorable interactions with the inhibitors, are depicted in the complexes according to the lowest energy structure from MD trajectory. (A) PMI-MDMX, (B)pDI-MDMX, (C) WK23-MDMX and (D) WW8-MDMX.

The inhibitor-residue interactions were computed by using the residue-based energy decomposition method[75] to evaluate the contributions of separate residues to the inhibitor association. The obtained detailed inhibitor-protein interaction spectra (Fig 6), analyses of hydrogen bonding interactions (Table 3) and the positions of the inhibitors relative to the key residues in the MDMX (Fig 7) were combined to probe the structure-affinity relationship of the binding compound.

**Table 3:** The hydrogen bonds of the key residues.

complex	Donor	Acceptor	Distance^*a*^/Å	Angle^*a*^/(°)	Freq.^*b*^/%
PMI-MDMX	Trp7′-NE1-HE1	Met53-O	2.83	149.25	92.31
pDI-MDMX	Trp7′-NE1-HE1	Met53-O	2.91	138.53	90.02
WK23-MDM2	WK23-N11-H4	Met53-O	2.83	157.25	97.04
WW8-MDMX	WW8-N8-H3	Met53-O	2.92	146.35	79.24

^a^The hydrogen bonds determined by the acceptor…donor atom distance of less than 3.5 Å and acceptor…H-donor angle of greater than 120 Å

^b^occupancy(%): to evaluate the stability and the strength of the hydrogen bond.

Fig 6A shows that the binding energies of nine residues Thr48, Met53, Ile60, Met61, Tyr66, His72, Val92, Tyr99 and Arg103 from MDMX to PMI are stronger than -1.30 kcal·mol^−1^. The interaction of Thr48 with PMI is about -1.31 kcal·mol^−1^, which may arise from the hydrophobic interaction between the CH groups of Thr48 and the alkyl of Leu10′ (Fig 7A). Among nine residues, the interaction energy of Met53 with PMI is the strongest (-4.87 kcal·mol^−1^). Analyses show that this interaction may arise from two contributions: (1) the CH-π interaction between the indole of Trp7′ and the alkyl of Met53, (2) the hydrogen bonding interaction between the atom NE1 of Trp7′ and the backbone oxygen O of Met53 (Table 2), with the distance of 2.83 Å and the occupancy of 92.31% between the corresponding oxygen and nitrogen atoms (Table 3). Structurally, the alkyls of Ile60 and Met61 are near the phenyl of Phe3′ and apt to generate the CH-π interactions, and their interaction intensities with PMI are -1.41 and -1.89 kcal·mol^−1^, respectively. The binding intensity of Tyr66 to PMI is -2.49 kcal·mol^−1^, which structurally agrees with a paralleled π-π interaction between the phenol of Tyr67 and the phenyl of Phe3′. The imidazole of His72 is in the vicinity of the phenyl of Phe3′ to generate a π-π interaction, which provides an energy contribution of -1.65 kcal·mol^−1^. The interaction intensity of Val92 with PMI is -2.81 kcal·mol^−1^, which mainly originates from the CH-π interaction between the alkyls of Val92 and the indole of Trp7′ (Fig 7A). According to Fig 6A, the residue Tyr99 also forms a very strong interaction with PMI (-4.47 kcal·mol^−1^), which is in good agreement with the CH-π interaction of the alkyl in Leu10′ with the phenol of Tyr99. Additionally, the positively charged residue Arg103 can generate strong polar interactions with the polar residues in the *C* terminus of PMI, which provides a contribution of -1.93 kcal·mol^−1^ to the PMI association.

By Comparison with the PMI-MDMX compound, it is observed from Figs 6B and 7B that eight residues in MDMX form strong interactions with pDI. These residues involve Lys50, Met53, Ile60, Met61, Tyr66, His72 Val92 and Tyr99. Except for Lys50, the interactions of the other seven residues with pDI are similar to those with PMI. The interaction energy of Lys50 with pDI is about -4.80 kcal·mol^−1^, which is mainly from the CH-CH interaction of CH group in Lys50 with Leu10′. Differently, the interactions of Met53, His72, Tyr99 and Arg103 with pDI decrease obviously, and the interaction of Thr48 with pDI disappears.

Figs 6C and 7C indicate that eight residues in MDMX produce strong interactions with WK23, and these residues are Met53, His54, Leu56, Gly57, Ile61, His72, Val92 and Tyr99. Compared to Fig 6A, the interaction modes of Met53, Ile61, His72, Val92 and Tyr99 with WK23 are similar to those with PMI and only interaction intensity changes. The interaction energy of His54 with WK23 is -1.34 kcal·mol^−1^, which mainly stems from the π-π interaction between the imidazole of His54 and the hydrophobic ring R2 of WK23. The alkyls of Leu56 and the CH group of Gly57 are near the ring R2 of WK23 to contribute the CH-π interaction energies of -1.55 and -2.2 kcal·mol^−1^ for the binding of WK23 to MDMX, respectively.

As seen in Figs 6D and 7D, the residues Lys50, Met53, His54 and His95 in MDMX produce obvious interactions with WW8. The interactions of Met53 and Gly57 with WW8 are similar to those with WK23. Structurally, the alkyls of Lys50 is close to the hydrophobic ring R4 of WW8 to generate a CH-π interaction, which contributes an energy of -1.38 kcal·mol^−1^. The energy of WW8 with His95 is -2.31 kcal·mol^−1^, which structurally agrees well with the π-π interaction of the hydrophobic ring in His95 with the rings R1 and R2 of WW8. The above results basically agree the previous studies[14, 30, 32].

Based on the above analyses, the interactions of separate residues in MDMX with pDI, WK23 and WW8 obviously change relative to those with PMI. The interactions of Tyr67, His72 and Tyr99 in MDMX with PMI are stronger than those with pDI, WK23 and WW8. These changes in the inhibitor-residue interactions correspondingly induce the changes of internal dynamics in MDMX. The previous cross-correlation analysis and RMSF values agree with the results reported here. In summary, the hydrophobic CH-CH, CH-π and π-π interactions drive the bindings of inhibitors to MDMX, thus optimization of these hydrophobic interactions is very helpful for successful designs of potent inhibitors targeting the p53-MDM2 interactions.

## Conclusion

60 ns molecular dynamics simulations were performed on four inhibitor-MDMX compounds to probe the effects of inhibitor bindings on the conformational changes of MDMX. The cross-correlation analysis and RMSF calculation based on MD trajectory show that the flexibility of MDMX binding cleft is large and the dynamic behavior of residues are different due to the presence of different structural inhibitors. The calculations of binding free energies by MM-PBSA and SIE method prove that van der Waals interaction drives the inhibitors binding to MDMX. The inhibitor-residue interactions were computed by using the residue-based energy decomposition method to obtain a detailed insight into the inhibitor-MDMX binding modes. The results indicate that the CH-CH, CH-π and π-π interactions between inhibitors and separate residues are the main forces controlling the inhibitor association to MDMX. This study can provide significant information on the structure-affinity relationship of the binding compound for the development of effective inhibitors targeting the p53-MDMX interaction.

## Supporting Information

S1 Fig RMSD values of the backbone atoms relative to the initial minimized structures as functions of simulation time. (DAT)

S2 Fig RMSF of Cα atoms in MDMX through the equilibrium phase of MD simulations. (DAT)

S3 Fig Cross-correlation matrices of the fluctuations of the coordinates for Cα atoms of MDMX around their mean positions during the equilibrium phase of simulations. (ZIP)

S4 Fig Comparison of the eigenvalues plotted against the corresponding eigenvector index obtained from the covariance matrix of Cα atoms constructed from the equilibrium of MD simulations. (DAT)

S5 Fig The interaction spectra of the separate residues of MDMX with the inhibitors. (ZIP)

S1 Table Calculation of binding free energy and energy component. (ZIP)
